# Malaria parasitemia and its association with liver function parameters, and lipid profile among malaria-infected adult patients in western Ethiopia: A comparative cross-sectional study

**DOI:** 10.1371/journal.pone.0345059

**Published:** 2026-03-20

**Authors:** Nigus Checkole, Waqtola Cheneke, Temam Ibrahim, Shiferaw Bekele, Birhane Teklay, Mebrahtu Tefera, Ataklti Gebretsadik, Bisrat Fikadu, Getachew Belay Kassahun

**Affiliations:** 1 Department of medical laboratory science, College of medicine and Health science, Adigrat University, Adigrat, Tigray, Ethiopia,; 2 Department of clinical chemistry, School of Medical Laboratory Science, Institute of Health science, Jimma University, Jimma, Oromia, Ethiopia,; 3 Department of Midwifery, College of medicine and Health science, Raya University, Maichew, Tigray, Ethiopia,; 4 Department of Pharmacy, College of medicine and Health Science, Adigrat University, Adigrat, Tigray, Ethiopia,; 5 Department of Medical Laboratory Sciences, College of Medicine and Health Science, Welkite University, Welkite, Ethiopia; Institute of Medical Research and Medicinal Plants Studies, CAMEROON

## Abstract

**Background:**

Malaria poses a public health problem because it manifests anemia, renal and liver dysfunction, Jaundice, and dyslipidemia. Therefore, the study aimed to assess malaria parasitemia and its association with liver function parameters and lipid profile in Assosa town, Ethiopia from December 23/2021 to March 4/2022.

**Methods:**

Institution-based comparative cross-sectional study was conducted among 302 study participants (151 study groups and 151comparison group) who were selected by consecutive sampling technique. Socio-demographic and clinical data were collected using structured questionnaires and entered using Epi Data version 4.6. Venous blood specimen was collected from all study individuals and tested for selected parameters by Cobas 311 automated clinical chemistry analyzer. Descriptive statistics, Pearson’s Chi-square, t-test, and ANOVA were used to assess the association using STATA software version 14.

**Results:**

A total of 302 study participants comprising 212(70.2%) males were included. Majority (70.86%) of malaria patients were infected with *P. falciparum* (The parasitic densities were reported with 30.46%, 46.36%, and 23.18% for low, moderate, and high parasitemia, respectively. The mean value of aspartate aminotransferase, alanine aminotransferase, alkaline phosphatase, and serum bilirubin was significantly higher in malaria patients than in apparently healthy controls (p < 0.001). However, the mean value of total cholesterol, high-density lipoprotein cholesterol, and low-density lipoprotein cholesterol was significantly lower in malaria patients than controls (P < 0.05).

**Conclusion and Recommendation:**

Liver function tests and lipid profile should be assessed for malaria infected individuals in order to prevent malaria complications.

## Introduction

Malaria is a vector-borne infectious disease caused by obligate intracellular parasites of the genus *Plasmodium*. It is transmitted by the bite of *Anopheles* mosquito mainly *Anopheles gambiae* and *Anopheles funestus* [1]. The four *Plasmodium* species, namely *Plasmodium falciparum* (*P. falciparum), Plasmodium vivax (P. vivax), Plasmodium ovale (P. ovale) and Plasmodium malariae (P. malariae),* are commonly cause malaria in humans [2]*.*

Globally, in 2023, there were nearly 263 million estimated malaria cases across 83 malaria-endemic countries. Since 2020, the number of estimated malaria cases has steadily increased, with most of this rise occurring in the African Region (89.7%), and 95% of malaria cases and 96% of deaths are concentrated in sub-Saharan Africa [3]. Ethiopia was a country primarily contributing to the increase in malaria cases between 2022 and 2023, and 75% of the country’s landmass is affected bythe disease. In addition, most transmission occurs between September and December following the main rainy season from June to August [4]. *Plasmodium falciparum* and P. *vivax* are the two dominant parasite species causing human malaria in Ethiopia with relative frequencies of about 62.8% and 37.2%, respectively [4,5].

*The most common signs and symptoms of malaria are fever, headache, malaise, weakness, gastrointestinal discomfort, neurological discomfort, back pain, and chills* [6]*. Hypoglycemia, anemia, renal dysfunction (acute kidney damage), jaundice, pulmonary edema, dyslipidemia, and liver dysfunction are common complications of severe malaria* [7].

The liver is a major organ that plays an important role in the development and replication of Plasmodium sporozoites into merozoites, which is crucial for the pathogenesis of malaria infection. Therefore, *infection of liver cells by the sporozoite stage of the malaria parasite can cause organ congestion, sinus block, and cellular inflammation* [8]*. Malaria-induced hepatocyte damage can significantly affect serum levels of the enzymes aspartate transaminase (AST), alanine transaminase (ALT), alkaline phosphatase (ALP), Bilirubin Total (BT), Bilirubin Direct (BD) and Indirect* (BID) [9].

Hepatocellular damage associated with severe and acute malaria parasite infections correspondingly impairs biochemical processes of lipid metabolism, leading to alterations of lipid and lipoprotein parameters [10]. Most of the endogenous lipoproteins in plasma come from the liver, which depends on the cellular integrity and functionality of the hepatocytes. Furthermore, Malaria parasites utilize cholesterol and phospholipids for survival in their human host [11].

Since the routine diagnostic procedure of malaria guideline in Ethiopia does not include an assessment of liver function tests and lipid profiles, most mortality and malaria complications were occurred due to liver dysfunction and dyslipidemia [12]. Data regarding association of serum lipid profile and liver function parameters withmalaria among infected individuals relative to the level of malaria parasitemia load, remains scarce for Ethiopian population. Therefore, this study was aimed to assess malaria parasitemia and its association with liver function parameters and lipid profiles among malaria-infected adult patients and non-infected comparators in western Ethiopia.

## Materials and methods

### Study design and period

Institutional based comparative cross- sectional study design was conducted from December 23/2021 to March 4/2022.

### Study area and population

The study was carried out in Assosa city, Beneshangul Gumuz regional state in western Ethiopia. Assosa is 679 kilometers far away from Addis Ababa, the capital city of Ethiopia, with altitude and longitude of 10^o^ 04‟ N 34 ^o^35‟E/10.06 ^o^N 34.517^o^ E, and elevation of 1570 meters above sea levels. According to the Assosa town municipality information center, the current total population of the town is 70,122 of which 35,271 are males. It has a maximum and minimum temperature of 40^o^C and 14^o^C, respectively. The average annual rainfall is 1,166 mm and the rain season which extends from April to November. Assosa is a malaria endemic area and owns three public health facilities, providing malaria diagnosis and treatment. The study was conducted in Assosa health center and Assosa General Hospital, which were randomly selected health facilities.

### Sample size determination

Sample size was calculated using double population proportion formula for comparison of two population means. The minimum sample size was calculated by Open Epi Version 3 open source calculator software and the mean and standard deviation of triglyceride(TG) among malaria patients and healthy controls (118.0 ± 61.3 vs 101.7 ± 36.8) was taken from a previous study conducted in Metema, Ethiopia in 2016 [13]. Two-sided confidence intervals (95%), 80% power were used to calculate sample size using the following formula giving a total sample size of 302.

Comparison of two means (sample size in each group)


$$\text{N}=\;({\text{S1}}^{2}+\;{\text{S2}}^{2})\;{\left(\text{Z}\alpha /\text{2}+\text{Z}\beta \right)}^{2}/\;{\left(\text{m1 }-\text{ m2}\right)}^{2}$$

Where, m1and S1^2^-are mean and variance of group 1 respectively.

m_2_and S_2_^2^- mean and variance of group 2 respectively and N = total sample size required

Power = 80% and the level of significance = 5%


$$\begin{array}{c} \text{N}=\;({\text{61}.\text{3}}^{2}\;+\;{\text{36}.\text{8}}^{2})\text{11}/\;{\left(\text{118 }-\text{ 101}.\text{7}\right)}^{2}\;=\text{ 302} \end{array}$$

$\text{N}=\;(\text{3757}.\text{69 }+\text{ 1354}.\text{2411}/\;{\left(\text{118 }-\text{ 101}.\text{7}\right)}^{\text{2}}=\text{ 302}$. Hence, the desired sample size for cases to controls allocation was one to one ratio (151 malaria positive and 151 health visitors) were included in the study*.*

### Sampling techniques

Consecutive sampling technique was used to recruit individuals until the required sample size had been reached.

### Eligibility Criteria

All smear positive for malaria pre-treatment adult patients and who were consented to participate in the study were included in malaria infected group and matched healthy visitors during the study period were included in the control group. However, Malaria patients with known diabetes, hypertensive, cardiac cases, HIV, hepatitis B or C virus, pregnant women, obese, and chronic liver disease were excluded from the study.

### Operational definitions

**Malaria parasitemia:** level of the asexual stage of malaria parasite inside of the host.

**Liver function parameters:** Tests commonly used to check liver function which includes Aspartate aminotransferase (AST), Alanine aminotransferase (ALT), Alkaline phosphatase (ALP), Bilirubin Total (BT), Bilirubin Direct (BD), and Bilirubin Indirect (BID).

**Lipid profile:** is a group of tests used to assess dyslipidemia that includes High density lipoprotein (HDL), Low density lipoprotein (LDL), Total Cholesterol (TC), and Triglyceride (TG).

**Acute febrile patients:** patient with fever of ≥ 37.5^o^c

**Study group**: are adult individuals who have malaria.

**Comparators:** are adult individuals who are apparently healthy and unlikely to share malaria.

### Data collection and laboratory investigation

The overall objectives of the study were informed to 3 Laboratory and nurse professionals for consecutive 2 days. A structured questionnaire was pre-tested in Jimma town to check its clarity and completeness before the actual data collection was started. After obtaining written informed consent from all study participants, malaria patients as cases and apparently healthy individuals as controls had been interviewed to obtain socio-demographic data. In addition, clinical data were taken by reviewing the medical records for participant’s eligibility.

About 4 mL of venous Blood specimen were collected by medical laboratory technologists into serum separator tube from each study participant. Two drops of whole blood was added on a frosted slide and thick blood film was made and stained using 10% Giemsa solution for malaria parasite detection and parasite density count. Moreover, thin blood film was also made and stained with Giemsa solution after fixation by absolute methanol for 30 second for *Plasmodium* species identification. Counting of parasite density was performed by well-trained laboratory technologists to the asexual parasite stages against 200 white blood cells (WBCs) in the thick blood film, by assuming an average of WBC count 8,000 cells/µL, and expressed as number of parasites per microliter of whole blood.

Hence: Number of malaria parasites per microliter of blood = number of parasites counted × 8,000 WBCs/ number of WBCs counted.

Case study participants below 1,000 parasites per μl, 1,000–10,000 parasites per μl, and above 10,000 parasites per μl of blood were considered low, moderate, and severe(high) infections, respective [14,15].The serum sample was separated by centrifugation at 4000 rpm for 5 minutes and stored in a freezer at a temperature of −20-(−30)°c prior to laboratory analysis. Biochemical tests such as lipid profile (HDL, TG, TC and LDL) and liver function parameters (AST, ALP, ALT, TB, and BD) were measured by cobas 311 fully automated clinical chemistry analyzer in Assosa General Hospital laboratory department.

### Quality assurance

Questionnaire was pre-tested for accuracy and consistency prior to the actual data collection. Completeness, correctness and clarity of the collected data were carefully checked on a regular basis. Experienced medical laboratory personnel’s were involved in the proper collection, processing, and transportation of the specimen. Standard Operating Procedures (SOPs) were followed to ensure the quality of laboratory testing. Both normal and pathologic quality controls were analyzed prior to sample analysis to ensure that the processes were followed correctly, proper instrument function, validity, and reliability. In addition, reagents for blood film preparation were checked for the expiration date, and quality control was done for every new batch. Finally, Samples were analyzed after both types of control were passed. About 10% of the total slides of study participants were randomly selected and re-examined by senior Medical Laboratory Technologist blinded to the first result.

### Statistical analysis

All the data from questionnaires were checked manually for completeness and comprehensibility before data analysis. Data were entered using EpiData version 4.6 and Statistical analysis was done using statistical software for data science (STATA) version 14. Descriptive statistics was performed and chi-square was used to determine relationships between categorical variables. Independent t-test was used to show mean differences of lipid profile and liver function parameters among malaria patients and control groups. Analysis of variance (ANOVA) was performed to know mean differences of lipid profile and liver function parameters among low, moderate, and high malaria parasitemia levels. The accepted level of significance for all statistical analysis used in this study was p < 0.05

### Ethical clearance

This study was conducted after ethical clearance was obtained from Jimma University Institute of Health Sciences School of Graduate Studies Ethical Review Board (JUIH-IRB). Letter of permission was obtained from chief executive directors of Assosa General Hospital and Assosa health Center. Finally, after written informed consent was obtained from each study participant’s actual data collection were started. In addition, the confidentiality of the study participants’ medical records and laboratory results was maintained by using unique identification codes instead of personal identifiers.

## Results

### Socio- demographic characteristics of study participants

A total of 302 study participants were included in this study comprising, 151 malaria confirmed patients (105 males) and 151 healthy control groups (107 males). The mean age ± SD of malaria patients and healthy controls were 29.47 ± 10.21 years (range 18–69 years) and 31.98 ± 9.67 years (range 18–63years), respectively. There was a male predominance in both case and control participants, and majority of the study participants were from rural residence, 98(64%) and 79(52%) for cases and controls, respectively (Table 1).

**Table 1:** Socio-demographic characteristics of the study participants(N = 302) at Assosa town public health institutions, Benishangul Gumuz region, western Ethiopia from December 23/2021 to March 4/2022.

Socio-demographic Variables	Malaria patients n = 151	Health controls n = 151	Total N = 302	p-value
Sex				
Male	105(69.54%)	107(70.86%)	212(70.2%)	
Female	46(30.46%)	44(29.14%)	90(29.8%)	
Total	151(100%)	151(100%)	302(100%)	0.8
Age				
18-27	86(56.95%)	58(38.41%)	144(47.68%)	
28-37	32(21.19%)	56(37.08%)	88(29.14%)	
38-47	24(15.89%)	27(17.88%)	51(16.89%)	
48-57	7(4.64%)	7(4.64%)	14(4.64%)	
>= 58	2(1.33%)	3(1.99%)	5(1.65%)	
Total	151(100%)	151(100%)	302(100%)	
Mean ±SD	29.47 ± 10.21	31.98 ± 9.67		0.16
Occupation				
Student	31(20.53%)	14((9.27%)	45(14.9%)	
House hold wife	29(19.21%)	25(16.56%)	54(17.88%)	
Farmer	37(24.5%)	41(27.15%)	78(25.83%)	
Government employer	19(12.58%)	40(26.49%)	59(19.54%)	
Private worker	14(9.27%)	8(5.3%)	22(7.28%)	
Merchants	12(7.95%)	12(7.95%)	24(7.95%	
Daily labor	9(5.96%)	11(7.28%)	20(6.62%)	
Total	151(100%)	151(100%)	302(100%)	0.013
Residence				
Urban	53(35.1%)	72(47.68%)	125(41.39%)	
Rural	98(64.9%)	79(52.32%)	177(58.61%)	
Total	151(100%)	151(100%)	302(100%)	0.026
Educational status				
No formal education	8(5.3%)	10(6.62%)	18(5.96%)	
1-8	51(33.77%)	39(25.83%)	90(29.80%)	
9-12	65(43.05%)	62(41.06%)	127(42.05%)	
> 12	27(17.88%)	40(26.49%)	67(22.19%)	
Total	151(100%)	151(100%)	302(100%)	0.22

### Clinical characteristics of malaria patients

**Fig 1:**
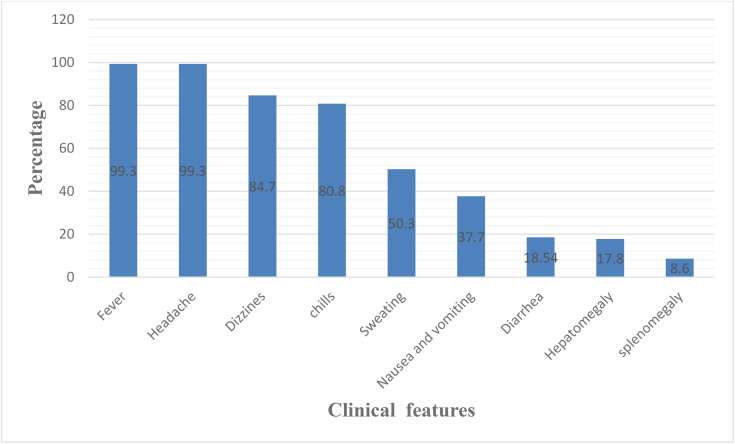
Clinical features of malaria infected adult patients (n=151) in Assosa town public health institutions, Benishangul Gumuz region, western Ethiopia from December 23/2021 to March 4/2022.

*Plasmodium falciparum* and *P. vivax* are the two dominant parasite species causing human malaria in Ethiopia [16]. The study indicated that a significant majority of malaria patients were infected with *P. falciparum* (70.86%), followed by *P. vivax* (25.82%), and mixed infections accounted for 3.32%. Among the case study participants, 30.46% were found to have low parasitemia, 46.36% had moderate parasitemia, and 23.18% exhibited high parasitemia loads. The main clinical symptoms and signs presented at the initial evaluation of malaria patients were fever (99.3%), headache (99.3%), dizziness (84.7%), chills (80.8%), sweating (50.7%) and the least is splenomegaly (8.6%) (Fig 1).

### Liver function parameters and lipid profile of study participants

The study showed statistically significant differences in liver function parameters and lipid profile between malaria infected and apparently healthy controls. AST, ALT, ALP, BT, BD, BID and TG were found to be significantly increased in malaria-infected patients compared to apparently healthy controls with mean ± SD (43.73 ± 18.15 vs 25.00 ± 11.17, 31.35 ± 21.64 vs 14.50 ± 7.29, 108.32 ± 41.69 vs 89.77 ± 35.51, 0.94 ± 0.49 vs 0.49 ± 0.32, 0.29 ± 0.22 vs 0.14 ± 0.17, 0.64 ± 0.37 vs 0.35 ± 0.24 (p < 0.001) and 138.14 ± 58.71 vs 123.87 ± 54.02 (p < 0.05) respectively. But, TC, HDL and LDL were found to be significantly decreased in malaria-infected patients compared to healthy controls with mean ± SD (105.75 ± 41.21 vs 165.45 ± 46.01, 19.19 ± 8.64 vs44.30 ± 9.45 and 48.91 ± 30.74 vs 88 ± 36.05 (P < 0.001) respectively (Table 2).

**Table 2:** Liver function parameters and lipid profile among malaria infected adult patients and control groups(N = 302) at Benishangul Gumuz region, Assosa town public health institutions, Western Ethiopia, from December 23/2021 to March 4/2022.

Lab Variables	Lab result for Cases (mean ± SD)	Lab result for Controls (mean ±SD	t-value	Mean difference	95%CI of mean difference	p-value
LFT						
AST	43.73 ± 18.15	25.00 ± 11.17	10.8	18.58	[15.31,22.14]	0.000*
ALT	31.35 ± 21.64	14.50 ± 7.29	9.06	16.85	[13.19,20.50]	0.000*
ALP	108.32 ± 41.69	89.77 ± 35.51	4.1	18.55	[9.77,27.32]	0.000*
BD	0.29 ± 0.22	0.14 ± 0.17	6.42	0.15	[0.103,0.195]	0.000*
BT	0.94 ± 0.49	0.49 ± 0.32	9.15	0.450	[0.347,0.537]	0.000*
BID	0.64 ± 0.37	0.35 ± 0.24	7.99	0.29	[0.220,0.363]	0.000*
Lipid profile						
TC	105.75 ± 41.21	165.45 ± 46.01	−11.8	−59.7	[-69.8,-49.8]	0.000*
TG	138.14 ± 58.71	123.87 ± 54.02	2.19	14.27	[1.49,27]	0.0287
HDL	19.19 ± 8.64	44.30 ± 9.45	−24	−25.11	[-27.17,-23]	0.000*
LDL	48.91 ± 30.74	88 ± 36.05	−10.2	−39.09	[-47.22,-32]	0.000*

* = Highly significant, Independent t-test was used and P-value ≤ 0.05 was considered as statistically significant.

Malaria-infected individuals were grouped according to their density of parasitemia as low, moderate and high degree of parasitemia. In the current study, the level of parasitemia has positive association with mean liver enzyme activities. Moderate and high parasitemia patients showed higher serum levels of liver enzyme activities compared to patients with low parasitemia. Serum bilirubin total also increased with increasing severity of parasitemia of malaria infection. However, the lipid profile (TC, HDL, and LDL) is inversely associated with parasitemia density (Table 3).

**Table 3:** Liver function parameters and lipid profile in different degree of malaria parasitemia among malaria infected adult patients (N = 151) at Benishangul Gumuz region, Assosa town public health institutions, Western Ethiopia, from December 23/2021 to March 4/2022.

Lab variables	Low(n = 46) Mean ±SD	Moderate(n = 70)Mean ±SD	High(n = 35) Mean ±SD	F- value	p-value
LFT					
AST	37.50 ± 14.17	38.80 ± 13.00	61.81 ± 20.14	32.04	0.000*
ALT	23.78 ± 14.98	26.42 ± 15.36	51.16 ± 27.56	25.62	0.000*
ALP	100.60 ± 33.6	106.05 ± 39.22	123.01 ± 52.33	3.15	0.045
DB	0.235 ± 0.17	0.276 ± 0.21	0.416 ± 0.270	7.48	0.008
TB	0.78 ± 0.38	0.84 ± 0.43	1.36 ± 0.52	20.92	0.000*
IDB	0.543 ± 0.315	0.560 ± 0.33	0.940 ± 0.396	17.11	0.000*
Lipid profile					
TC	117.46 ± 43.12	107.36 ± 41.60	87.15 ± 31.17	5.83	0.0037
TG	136.34 ± 56.70	140.12 ± 60.46	136.59 ± 59.33	0.07	0.930
HDL	22.15 ± 10.94	19.56 ± 7.70	14.56 ± 4.10	8.57	0.0003*
LDL	57.30 ± 31.57	49.63 ± 30.90	36.47 ± 25.67	4.83	0.0093

* = Highly significant and ANOVA was used and P-value ≤ 0.05 was considered as statistical significant.

According to the current result (Table 3), there is a positive statistically significant differences among the liver parameters and parasitemia load (p < 0.05). In addition, a negative statistical significant differences (P < 0.05) was observed between density of malaria and lipid profile (TC, HDL, LDL). But, there is no statistically significant differences (P > 0.05) between parasitemia density and triglyceride level (Table 3).

## Discussion

Malaria is one of the greatest public health challenges worldwide, especially in tropical and sub-tropical countries. The liver, red blood cells, and lipid profiles undergo significant changes during the early stages of infection [13,17]. The result of the study showed a significant statistical difference (p < 0.001) between malaria infected cases and apparently healthy control study participants in their mean values of serum ALT, AST, ALP, BT, BD and BID. The mean values were significantly higher in malaria patients than in apparently healthy controls. This finding is consistent with previous studies conducted in Australia, Sierra Leone, Sudan, Nigeria, and India [18–21]. The observed increment in serum liver enzymes (AST, ALT and ALP) could be due to leakage from hepatocyte cells injured by the development of autoimmunity and/or abnormal cell activation induced by the parasite during the infection process [22]. In addition, the higher level of bilirubin level could be due to the presence of extra-hepatic hemolysis as the primary cause of jaundice or due to bile duct obstruction or damage to the hepatocellular structure by infection of *Plasmodium* parasites [23].

The study appreciated serum AST, ALT, ALP, and Bilirubin parameters were found to be positive relationship with parasitemia load (p < 0.05). This was in line with previous studies conducted in Nigeria and Yemen [9,12,24]. However, mean value of lipid profiles (HDL, TC and LDL) were decreased with increased parasitemia load. This was in line with studies conducted in Gabon, Saudi Arabia, Brazil, and Metema Ethiopia [13,25–27]. This could be due to plasmodium parasites that can control and alter the host’s lipid metabolic pathways [10,25,26,28].

Mean value of serum TG among cases were significantly higher than controls; but serum TC, HDL, and LDL were significantly lower in malaria-infected patients. This result was in line with studies conducted in India, Saudi Arabia and Gabon [26,27,29]. This could be due to cholesterol being synthesized in the liver, which is a key site in malaria pathogenesis and despite of the fact that the parasites grow and multiply by consuming the host’s nutrients, they are unable to synthesize a large portion of their own lipids [27]. The Significant elevation of TG could be due to host-related reactions associated with acute-phase reactions. Moreover, the source of variation with our study could be due to differences in diet, genetic variability, different in malaria diagnostic techniques, parasite density and analytical and quality control factors [26]. Furthermore, a systematic review and meta-analysis supported our findings, which found that TC, HDL, and LDL concentrations were lower in malaria patients compared to apparently healthy controls(p < 0.05) [30].

A study conducted in Nigeria reported that the mean value of serum TC and TG of malaria-infected patients and health comparators had no significant differences, which disagrees with the current study findings. However, It in lines with mean value of serum LDL and HDL [31,32]. The agreement could be due to plasmodium parasite use of host cholesterol and phospholipids, and detrimental effects of parasites on the rate of HDL and LDL synthesis [33]. The source of disagreement of the findings with our findings could be due to small sample size used in the previous studies, parasite density, lifestyle difference, analytical and quality control factors, and eligibility criteria.

## Conclusion and recommendations

Malaria patients had significant changes in liver function parameters and lipid profile. This suggests that malaria is one of the causes of liver function and lipid profile abnormalities. The study recommends liver function tests and lipid profiles should be performed as standard diagnostic tests among malaria diagnosed individual to reduce malaria complications. Furthermore, researchers are needed to conduct a longitudinal study for better understanding of the influence of malaria parasite density on liver function tests and lipid profile.
